# Wearable Electrochemical Sensors in Parkinson’s Disease

**DOI:** 10.3390/s22030951

**Published:** 2022-01-26

**Authors:** Francesco Asci, Giorgio Vivacqua, Alessandro Zampogna, Valentina D’Onofrio, Adolfo Mazzeo, Antonio Suppa

**Affiliations:** 1 IRCCS Neuromed, 86077 Pozzilli, IS, Italy; francesco.asci@uniroma1.it; 2 Integrated Research Center (PRAAB), Campus Biomedico University of Roma, Via Alvaro del Portillo 21, 00125 Rome, RM, Italy; g.vivacqua@unicampus.it; 3 Department of Human Neurosciences, Sapienza University of Rome, 00185 Rome, RM, Italy; alessandro.zampogna@uniroma1.it (A.Z.); valentina.donofrio@uniroma1.it (V.D.); adolfo.mazzeo@uniroma1.it (A.M.)

**Keywords:** Parkinson’s disease, L-Dopa, biosensors, wearable sensors, electrochemical monitoring

## Abstract

Parkinson’s disease (PD) is a neurodegenerative disorder associated with widespread aggregation of α-synuclein and dopaminergic neuronal loss in the substantia nigra pars compacta. As a result, striatal dopaminergic denervation leads to functional changes in the cortico-basal-ganglia-thalamo-cortical loop, which in turn cause most of the parkinsonian signs and symptoms. Despite tremendous advances in the field in the last two decades, the overall management (i.e., diagnosis and follow-up) of patients with PD remains largely based on clinical procedures. Accordingly, a relevant advance in the field would require the development of innovative biomarkers for PD. Recently, the development of miniaturized electrochemical sensors has opened new opportunities in the clinical management of PD thanks to wearable devices able to detect specific biological molecules from various body fluids. We here first summarize the main wearable electrochemical technologies currently available and their possible use as medical devices. Then, we critically discuss the possible strengths and weaknesses of wearable electrochemical devices in the management of chronic diseases including PD. Finally, we speculate about possible future applications of wearable electrochemical sensors in PD, such as the attractive opportunity for personalized closed-loop therapeutic approaches.

## 1. Introduction

Parkinson’s disease (PD) is the second most common neurodegenerative disorder and one of the most relevant in terms of future global burden of diseases, as shown by the steep rise in its prevalence over the past two decades [1]. To date, due to the lack of reliable biomarkers, the clinical diagnosis and follow-up of PD still rely on neurological examination with dedicated clinical scales [2,3]. In addition, the therapeutic management of PD, which mainly consists of the adjustment of L-Dopa therapy, is commonly based on the clinical evaluation of motor and non-motor symptoms. Hence, the development of new biomarkers for the diagnosis and follow-up of PD would be a relevant advance in the field.

In recent years, the development of wearable electrochemical sensing platforms for the detection of various biological fluids has provided new opportunities for the clinical management of patients affected by chronic disorders, including PD. According to International Union of Pure and Applied Chemistry (IUPAC) recommendations, an electrochemical biosensor is a self-contained integrated device able to provide specific quantitative or semi-quantitative analytical information, by using a biological recognition element (i.e., biochemical receptor), retained in direct spatial contact with an electrochemical transduction element [4]. Among chemical platforms, those based on microneedles (MNs) have preliminarily demonstrated high reliability due to the fast, real-time, and minimally invasive recording of several medically relevant molecules.

The present manuscript is organized into four main sections. In the first section, we summarize the main clinical, pathophysiological and therapeutic issues in the management of patients with PD. Furthermore, we discuss how advanced electrochemical biosensing systems would contribute to the instrumental diagnosis and monitoring of PD. In the second paragraph, following some historical notes, we review the state of the art of electrochemical biosensors, describing types, materials, chemical reactions and specific molecules detected. In the third section, we focus on the existing electrochemical biosensing tools for detecting medically relevant biological molecules in PD, including alpha-synuclein and dopamine. Lastly, the final paragraph discusses possible perspectives for integrated clinical management of PD.

## 2. Hunting Biomarkers for Diagnosis and Follow-Up in PD

Patients affected by PD manifest a variable association of motor symptoms, including bradykinesia, rigidity, and tremor, as well as non-motor symptoms, such as cognitive decline, autonomic dysfunction, depression, and pain [1]. Unfortunately, during the preclinical stage or also at an early stage of the disease, patients may report unclear symptoms which are very difficult to interpret. Additionally, the assessment of PD patients still relies on neurological examination with the aid of dedicated clinical scales for disease staging, including the Hoehn and Yahr (H & Y) scale, and for classifying parkinsonian symptoms such as the Unified Parkinson’s Disease Rating Scale (UPDRS) [2,3]. Hence, characterizing a reliable disease biomarker would help clinicians to achieve an objective diagnosis of PD in vivo and at early stages. Currently, the confirmed diagnosis of PD can be made only by analyzing post-mortem brain samples [5]. However, the molecular pathology underlying PD is also still largely unknown, though it has been related to aberrant α-synuclein aggregations, mitochondrial and lysosomal dysfunctions, and neuroinflammation [5]. The degeneration of dopaminergic neurons in the substantia nigra-pars compacta represents the cardinal neuropathological feature of PD, resulting in a functional imbalance in the direct and indirect pathways of the basal ganglia, which leads, in turn, to the cardinal parkinsonian motor symptoms [1]. Less is known, however, about the pathophysiological and neuroanatomical circuits underlying non-motor symptoms, which are also poorly responsive to dopaminergic therapy. Experimental and clinical evidence demonstrated the crucial role of neurotransmitters other than dopamine in the pathophysiology of specific motor and non-motor symptoms in PD [6]. Given the uncertainty about the actual diagnosis and pathophysiological mechanisms, the development of innovative disease biomarkers relying on safe, easy, and cheap methodologies would be a major achievement in PD. Hence, minimally invasive wearable biosensors able to extract and analyze relevant biological compounds from samples would allow achieving an objective diagnosis in PD.

**Figure 1:**
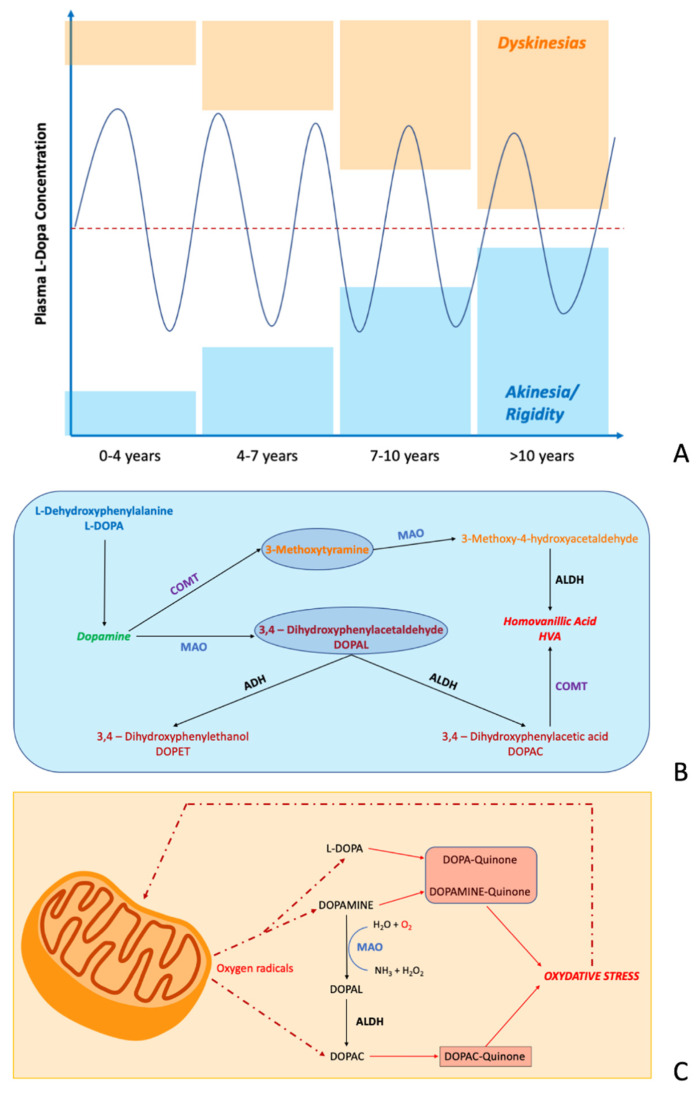
The metabolism of dopamine in Parkinson’s disease. (**A**) Progressive narrowing of the L-DOPA therapeutic window during disease progression, with the occurrence of an increased risk of developing dyskinesia (orange boxes) and motor blocks characterized by akinesia and rigidity (blue boxes). (**B**) Dopamine catabolism regulated by different enzymes including Mono-Amine-Oxidase (MAO), Catechol-O-Methyl Transferase (COMT), Aldehyde Hydrogenase (ADH) and Aldehyde De-Hydrogenase (ALDH). (**C**) Oxidation process of dopamine and L-DOPA in DOPA-quinone and Dopamine-quinone by oxygen radicals produced during mitochondrial respiration.

Regarding therapeutic strategies in PD, current pharmacological approaches are primarily aimed at replacing the nigrostriatal dopamine precursors through the administration of exogenous L-Dopa. Accordingly, the best medical treatment in PD patients consists of the administration of oral L-Dopa tablets, according to personalized timing and dosing [1,7]. Following the start of the dopaminergic therapy, early-stage patients experience a dramatic improvement of symptoms, justifying the definition of “miracle drug” used when L-dopa was first discovered in the late 1960s [7,8]. However, over the disease’s progression, the increasing loss of dopaminergic neurons and the chronic exposure to dopaminergic drugs lead to decayed long-duration and increased short-duration responses to L-Dopa (i.e., abnormal dopamine synthesis, storage, release, and buffering) [9]. As a result of these changes, patients manifest motor complications, such as wearing-off phenomenon, motor fluctuations [1], unpredictable OFF times [10,11], and L-Dopa-induced dyskinesias (LIDs) [12], which all depend on the fluctuation of L-Dopa plasmatic levels [9,13,14]. The progressive narrowing of the L-Dopa therapeutic window leads patients to take higher drug doses, also increasing the administration intervals. In addition, to further increase the complexity of pharmacologic management of PD patients, the intersubject variability of response to L-Dopa therapy is rather high. Accordingly, the development of minimally-invasive wearable biosensors able to achieve real-time monitoring of the L-Dopa plasmatic concentration would be a relevant breakthrough in the field of PD, especially in the advanced stages of the disease [15]. See also Figure 1.

## 3. Electrochemical Biosensors

Historically, the first pioneering attempts to produce microdevices for human application started with the development of MNs to improve the transdermal delivery of specific drugs [16]. Since then, several clinical trials have included miniaturized devices for the treatment of diabetes mellitus [17], cancer [18] and neuropathic pain [19]. Although first conceptualized in the 1970s, it was only over the course of the late 1990s that the perspective began to change from the administration of drugs to the detection of relevant disease biomarkers, including glucose [20], lactate [21], alcohol [22], biogenic amines (i.e., dopamine, norepinephrine, and serotonin) [23,24] and L-Dopa [25]. Over the last few years, researchers in bioengineering have designed more advanced biosensors aimed at detecting medically relevant biomolecules from internal (i.e., blood and interstitial fluid—ISF) and, to a lesser extent, external fluids (i.e., saliva, sweat, tear and urine) [26]. Accordingly, several types of chemical sensing platforms have been produced and released for medical purposes [15,25,27,28,29,30]. Specifically, among chemical platforms, the MN biosensors have deserved the largest funding due to the fast, real-time, reliable and minimally invasive recording of several biological molecules, with potential application as wearable, miniaturized, and portable devices [15,31,32]. Moreover, among MNs, those able to detect molecules from ISF received the greatest interest, since ISF has molecular concentrations comparable to blood [33].

MN biosensors used in research settings [15,31,32] consist of micron-sized arrays with a length of 150–1500 μm and a width of 50–250 μm, arranged on a miniature patch in a specific order. Concerning the measurement methods, technologies included in MNs rely on four main principles: voltammetry, amperometry, potentiometry and impedance. Voltammetry sensors calculate the concentration of specific molecules by measuring changes in current induced by applying variable electrical potentials (i.e., sweep, triangular, cyclic). By contrast, amperometry biosensors detect specific molecules by measuring the overall current in a circuit produced by applying a uniform electric potential (i.e., chronoamperometry). Furthermore, potentiometry sensors detect molecules by calculating the variation in potentials induced by applying a constant electric current. Lastly, impedance sensors works by measuring electric impedance resulting from applying alternating electric potentials [34]. Concerning the sampling methods, biological materials can be collected from the ISF by means of negative pressure (i.e., vacuum), capillary force, or material absorption. MNs based on negative pressure allow collecting a large volume of blood, being, however, more traumatic. MNs are currently manufactured using a variety of materials, including polymers, glass, ceramic, and metal with different shapes [30]. Several fabrication techniques have been proposed, including dry or wet etching, ion etching, laser ablation, photolithography, 3D printing, and micro-molding showing high accuracy [30].

Depending on their structure, MN biosensors can be divided into four main types: solid, hollow, coated, and dissolving. Solid MNs are projected and designed in microchannels which are placed on the skin surface, in a layer between the epidermis and dermis. Solid MN biosensors are produced using silicon, titanium, stainless steel, glass, ceramics, and nickel-iron, which are variously combined to define a pre-treated surface interacting with the skin. The microchannels allow medically relevant molecules to be sampled from the ISF through the dermis layer [30]. Hollow MNs are equipped with a dedicated reservoir for collecting the biological samples [28,30]. Devices are fabricated with silicon, metal, polymer, ceramic, and glass, which have allowed manufacturers to considerably decrease the micron size of the hypodermic needles. Compared to other MNs, hollow MNs can collect a large number of molecules for further analysis [30]. Coated MNs contain water-soluble biomolecules on their tips for capturing relevant biological compounds. Coated MNs are made of metal or silicon materials that confer adequate mechanical strength, rendering these types of electrochemical sensors stable for a long time and thus very relevant for biosensor-based analysis. Lastly, dissolving MNs are made of carbohydrates, PVA, PVP, PLA, PLGA, and sodium carboxymethyl cellulose. Over the last year, dissolving MNs have proved to be very suitable for biosensing purposes, due to the considerable patient compliance resulting from the non-invasiveness of analytic procedures [30]. As a result, each MN type has intrinsic strengths and limitations, although dissolving MNs would be promising given that they are minimally invasive and may provide an optimal administration route for pharmacologic compounds.

Depending on where the sample is analyzed, MNs are classified into the “Off device”, the “On device” and finally the “On MN” [28]. The “Off device” samples the ISF, but further analysis is performed in the central lab. The “On device” presents a miniaturized analyzer that is integrated and connected to the MN device for further analysis. Finally, the “On MN” is able to collect and analyze the biomarker directly in vivo [28]. Given that the “Off device” is affected by insufficient sampling and time-consuming follow-up procedures, the last two devices allow achieving the highest advantages in terms of real-life application and overall costs, being able to perform the analysis in situ [28]. Usually, hollow MNs present an attached sensor to the base or into the lumen and work as an “On device”, whereas solid MNs work as “On MN” given that their surfaces work as biosensors [28].

The functioning principles of electrochemical biosensors rely on different principles including electrochemical, optical, magnetic, and paper-based analysis [30]. However, owing to the miniaturization, highly scalable fabrication, rapid, inexpensive, low-power consumption requirements, and easier deployment, the MNs based on biochemical analysis, including the colorimetry, sandwich immunoassay, enzyme-labeled electrochemical immunoassay, nucleic acid recognition and enzymatic/nonenzymic electrochemistry, have been mostly investigated [28,30]. Among the electrochemical devices, those based on the enzyme and antibody/antigen are mostly used [28]. Overall, MNs offer high advantages in terms of rapidity, ease of use, and reliability of results compared to other wearable chemical sensing platforms [30]. See Table 1 for a detailed schematization of wearable electrochemical biosensors.

## 4. Electrochemical Biosensors in PD

Concerning the development of wearable, minimally invasive MN devices for early diagnosis of PD, current research has focused on the detection of α-synuclein from blood samples through a graphene oxide cysteamine-based electrochemical sensor [35]. Another biological compound that has been proposed in neurodegenerative diseases, including PD, is hydrogen peroxide (H_2_O_2_), a reactive oxygen species implicated in neurotoxicity processes. H_2_O_2_ can be extracted and analyzed from human serum and urine samples, but only in a laboratory setting [29]. However, although H_2_O_2_ and ROS are crucially involved in the oxidative stress underlying the pathogenic mechanisms of neurodegenerative diseases, they cannot be considered disease biomarkers useful for current clinical practice. So far, no one has developed wearable biosensor devices to detect specific PD biomarkers, including α-synuclein [36].

**Figure 2:**
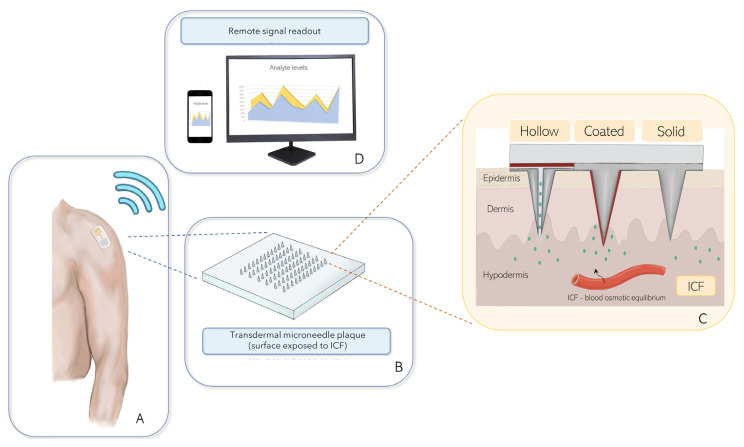
Possible application of a wearable transdermal MN-based biosensing system for the remote comprehensive monitoring of dopamine in patients with Parkinson’s disease. (**A**) Example of a wearable MN biosensing patch; (**B**) Simplified transdermal microneedle plaque; (**C**) Types of MN-based biosensors for detecting dopamine from the interstitial fluid; (**D**) Remote signal readout for telemedicine purposes.

Concerning the development of biosensors to detect L-Dopa, high-performance liquid chromatography has been proven to reliably detect the plasmatic concentration of L-Dopa, also correlating with the severity of motor symptoms [37]. However, chromatography cannot be applied in a free-living scenario since it is a time-consuming procedure with high costs [38]. Accordingly, several researchers are developing electrochemical devices for timely in vitro measurements of L-Dopa. These studies have prompted the production of wearable miniaturized devices for in vivo analysis of L-Dopa [15,30]. For these purposes, bioengineers have mainly focused on wearable biosensing MNs based on electrochemical analysis, due to the properties of L-Dopa. Goud et al. [15] have provided the first pioneering observation of a reliable MN device able to detect L-Dopa. More in detail, they have proposed a new orthogonal, wearable, minimally invasive, electrochemical/biocatalytic MN biosensor for the continuous monitoring of L-Dopa and of the oxidative metabolite Dopa-quinone [15]. However, MNs aiming to detect L-Dopa or its metabolites have to cope with a number of interfering species present at relatively high concentrations in the human brain and blood, including uric acid, serotonin and ascorbic acid. For instance, to limit the interfering effect of ascorbic acid during continuous in vivo recordings, considerable efforts have been undertaken to fabricate highly selective membrane coatings, including naflon and poly-o-phenylenediamine [39]. In addition, Jayaprakash et al. have used a cetyl pyridinium bromide (CPB)-modified carbon paste electrode (CPBMCPE) for accurate detection of dopamine and uric acid from relevant biological fluids [40]. These authors have demonstrated that CPB constitutes a relevant site for direct electron transfer to the CPBMCPE interface, increasing the carbon-electrode sensitivity for dopamine detection much more than other surfactants [40]. Accordingly, future research is required to verify whether MN biosensing devices for detecting L-Dopa and dopamine would be able to provide continuous analysis of such biological compound in ISF, also taking into account drawbacks related to interfering species such as ascorbic acid [39]. This methodology is promising because it allows for the monitoring of blood concentrations of L-Dopa and contextually of the oxidative products of L-Dopa metabolism, which are strategically helpful for clinicians, given that several drugs administered with L-Dopa target specific enzymes involved in the modulation of dopamine metabolism, such as monoamine-oxidase type B inhibitors. However, although electrochemical biosensors are able to precisely measure the plasmatic level of L-Dopa as well as its metabolites, even at micromolar concentrations, the relationship between the L-Dopa plasmatic levels and the central pharmacological action of the drug is rather complex and difficult to predict [41]. A further interesting approach to detect L-Dopa in PD patients without sampling blood or ISF with microneedles consists of wearable electrochemical platforms based on biosensor devices able to detect L-Dopa from other external fluids [25]. Basically, these electrochemical platforms work by catalyzing redox reactions in which dopamine is oxidized to dopamine-o-quinone by applying voltage [42]. However, to date, only one study has shown that a wearable miniaturized electrochemical fingertip device can detect L-Dopa from sweat, with reliable results [25]. Further studies are required to verify whether similar devices could be applied to detect L-Dopa from other body fluids, including saliva and tears. See Table 2 for a detailed report of published studies on the field of electrochemical sensors in PD. See also Figure 2 for a schematic diagram of a wearable MN electrochemical biosensor useful for monitoring dopamine in PD.

## 5. Clinical Prospects in PD

The diagnosis of PD requires objective disease biomarkers [2,3]. Over the last few years, several authors have focused on the research of biological biomarkers, including α-synuclein from cerebrospinal fluid and blood samples, in PD. However, these methodologies are mostly based on invasive and expensive devices. To overcome this issue, a few studies have begun to develop wearable minimally invasive devices for detecting α-synuclein in the effort to make an early PD diagnosis. By using graphene oxide cysteamine-based electrochemical sensors, preliminary data have shown that the methodology used should be improved. Furthermore, the diagnostic potential of α-synuclein is currently under debate and several studies are still ongoing to understand the molecular features of α-synuclein aggregates in additional biological fluids, including blood and saliva [52,53]. Finally, real-time monitoring of α-synuclein aggregates from external body fluids would clarify the clinico-pathological progression of the disease, and thus would verify the efficacy of disease-modifying therapies.

Patients with PD undergo progressive worsening of motor and non-motor symptoms over the course of the disease. The severity of PD-related symptoms has been related to the progressive loss of dopaminergic nigrostriatal neurons, from 30–65% at early-stage to more than 85% at advanced-stage disease. Further complicating this issue, it has been observed that the pharmacokinetic effects of L-Dopa become non-linear in a more advanced stage of the disease, being responsible for several complications. Although early-stage patients benefit from oral L-Dopa administration, more advanced patients experience a wearing-off phenomenon, motor fluctuations [1], unpredictable OFF times [10,11], and LIDs [12], which all depend on the fluctuation of L-Dopa plasmatic levels [9,13,14]. Hence, advanced-stage PD patients would benefit from advanced therapies designed to overcome the drawbacks of L-Dopa pharmacokinetic effects [54] by continuous infusion of the drug. Among these, the implantation of Levodopa/carbidopa intestinal gel (LCIG) represents a milestone of advanced-stage PD treatment, which would be implemented when oral dopaminergic therapy has not given satisfactory results [54]. However, although long-term treatment with LCIG demonstrated sustained significant and clinically beneficial reductions in OFF time [55], it may also cause “bodily discomfort” to patients due to the implantation of an infusion pump into the duodenum [56]. A second relevant treatment for advanced-stage PD is represented by the subcutaneous L-Dopa/carbidopa infusion, which has been developed to provide a minimally invasive sub-continuous infusion in a safe manner [57]. Preliminary data showed that this advanced therapy would be useful in PD patients experiencing severe motor fluctuations and prolonged OFF time [57]. However, in the case of infusional dopaminergic therapies, administration rates of L-Dopa need to be strictly personalized to each individual patient. Moreover, since these solutions are invasive and restricted to selected cohorts of patients, new non-invasive therapeutic approaches are necessary to improve the clinical management of advanced PD patients. Hence, a reliable biosensor able to regulate the administration of the drug based on the real-time assessment of motor symptoms and L-Dopa plasmatic concentration would be a true breakthrough for the management of PD, although, to date, an integrated sensing/infusing microdevice is not available yet. Minimally invasive, portable, and miniaturized biosensor platforms able to detect L-Dopa would be of increasing interest for future therapeutic implications in PD. In addition, the long-term, minimally invasive monitoring of L-Dopa plasmatic levels by means of dedicated electrochemical biosensors could help clinicians to predict motor fluctuations and LIDs in chronically treated patients. This advanced monitoring would offer a great opportunity to individualize pharmacological regimens, thus avoiding drug-related complications. The natural consequence of this achievement will be the large-scale production and marketing of specific closed-loop systems which will work by automatically regulating the amount of L-Dopa injected, based on specific plasmatic L-Dopa ranges, within the optimal therapeutic window. To further optimize the efficacy of this dual approach, closed-loop systems would be further improved by integrating new technologies based on specific injection systems able to assure linear profiles of L-Dopa plasmatic concentration [58].

A further relevant prospect for future application of biosensors will come from the integration of electrochemical sensing systems with new unobtrusive body-worn inertial sensors. Wearable inertial sensors, including tri-axial accelerometers and gyroscopes, are light and small devices that can be placed on different body segments, depending on the motor task to be examined. Several studies have demonstrated that inertial sensors can provide relevant information about motor performances by recording spatio-temporal and 3D kinematic data of body spatial orientation and motion [59]. More in detail, wearable inertial sensors have been largely used to objectively assess motor symptoms and quantify the disease severity in PD [60,61,62,63]. Moreover, advances in microelectronics have led to the production of small, flexible, comfortable devices that can be integrated into clothing (“e-textile”) [59]. The combination of electrochemical systems sensing L-Dopa plasma levels with new wearable inertial sensors would therefore allow for real-time correlation between the plasmatic levels of L-Dopa and the severity of motor symptoms. This approach would optimize real-time therapeutic strategies by tailoring pharmacological schedules to the current severity of motor symptoms and activity-dependent changes in free-living settings. We speculate that such integrating technology would further improve the reliability of closed-loop adaptive systems.

Finally, future studies will combine wearable sensing systems with digital signal readout and smartphone-based integrated systems for the simultaneous detection of several relevant biomolecules aiming to better characterize the clinical and neuropathological progression of PD. We suggest that future research in the field will pave the way to wearable, miniaturized electrochemical devices able to communicate directly with caregivers and general practitioners through wireless connection for a better patient-centered therapeutic approach [64]. By supporting integrated and global health care management, Internet of Things (IoT) resources will promote the pervasive use of multimodal wearable devices for innovative telemedicine approaches in patients with PD [65,66].

In conclusion, in this manuscript, we have summarized the state of the art of electrochemical biosensors currently available for the objective detection of specific molecules relevant for the instrumental diagnosis and follow-up of PD. We have also speculated about possible future applications of electrochemical devices for integrated management of patients with PD.

## Figures and Tables

**Table 1:** Electrochemical biosensors: types, materials, and methods.

Types of Biosensors	Measurement Methods	Sampling Methods	Fabrication Material	Fabrication Methods
Platforms	Impedance Potentiometry Amperometry (enzymatic) Voltammetry (non-enzymatic)	Vacuum Capillary Swelling	Polymers Silicon	Laser ablation Laser cutting
Solid MNs	Impedance Potentiometry Amperometry (enzymatic) Voltammetry (non-enzymatic)	Compression Absorption Vacuum	Silicon Ceramics Glass Metals	Laser ablation Laser cutting Casting Electroplating Lithography Wet and dry etching methods Metal injection molding Micromolding Two photon polymerization
Coated MNs	Impedance Potentiometry Amperometry (enzymatic) Voltammetry (non-enzymatic)	Capillary	Silicon Metals	Micromolding Dip coating Spray coating Layer-by-layer manufacturing
Dissolving MNs	Impedance Potentiometry Amperometry (enzymatic) Voltammetry (non-enzymatic)	Swelling	Cellulose Carbohydrates Sodium carboxymethyl	Mold based techniques Drawing lithography UV assisted fabrication Heat Droplet air blowing Fused deposition modeling Atomized spray process
Hollow MNs	Impedance Potentiometry Amperometry (enzymatic) Voltammetry (non-enzymatic)	Capillary Vacuum	Silicon Ceramics Polymers Glass Metals	MEMS Deep reactive ion etching Photolithographic Micromachining Pipette technique Deep X-ray lithography

MN: microneedle.

**Table 2:** Electrochemical biosensors in Parkinson’s disease: main achievement.

Authors	Year	Type of Biosensor	Chemical Process	Experiment	Fluid	Biomarker	LODs
Ali et al. [43]	2007	poly (anilineboronic acid)/carbon nanotube composit	Dopamine oxidation	In vitro	Blood	Dopamine	-
Bai and Jiang [44]	2013	Copper sulfide-decorated reduced graphene oxide composites	CuS/RGO composite-based reaction	In vitro	-	H2O2	-
Xu et al. [35]	2015	Cysteamine-graphene modified gold electrode nanocomposites	Carboxylic acid-induced covalent attachment	In vitro	Serum	α-synuclein	1.2 pM
Wang et al. [45]	2015	Gold Fe_3_O_4_ Platinum Graphene-based nanocomposites	Catalytic reaction of Pt RGO/AuFe_3_O_4_-GCE	In vitro	Normal and tumor cells	H_2_O_2_	0.1 μM
Oh et al. [46]	2017	Organic field-effect-transistor-type nonenzymatic biosensor	Dopamine oxidation	In vivo	ISF	L-Dopa	10 pM
Goud et al. [15]	2019	Orthogonal electrochemical/ biocatalytic hollow MN	Dopamine oxidation	In vivo/In vitro	ISF	L-Dopa	-
Nguyen et al. [47]	2019	Platinum-based nanocomposite	Glutamate oxidation	In vitro	Spinal cord sample	Glutamate	0.2–0.5 μM
Aziz et al. 2019 [48]	2019	LDHs and graphene-based nanocomposite	Dopamine oxidation	In vitro	Living cells	Dopamine	2.0 nM
Dong et al. [49]	2020	5-(1,2-dithiolan-3-yl)-N-(4-(4,4,5,5-tetramethyl-1,3,2-dioxaborolan-2-yl) phenyl) pent-anamide	One-step amide reaction	In vitro	Blood	H_2_O_2_	0.02 μM
Chang et al. [50]	2021	Nanobiosensor integrated with solid-phase microextractiontechnique	Dopamine oxidation	In vitro	Cytoplasm of single living cell	Dopamine	10 pM
Moon et al. [25]	2021	Wearable electrochemical platform	L-Dopa oxidation	In vivo/In vitro	Sweat/Blood	L-Dopa	-
Shi et al. [51]	2021	N-doped carbon nanorods and Au nanoparticles based biosensor	Dopamine oxidation	In vivo	Serum	Dopamine	-
Kudur-Jayaprakash et al. [40]	2021	Cetyl pyridinium bromide (CPB) modified carbon paste electrode (CPBMCPE) biosensor	Dopamine/Uric Acid-Voltammetric oxidation	In vivo	Urine	Dopamine/Uric Acid	38–42 μM

LOD: limit of detection.

## Data Availability

Not applicable.
